# Women in neurosurgery aim for recognition of merit, not tokenism: insights from an Italian survey

**DOI:** 10.3389/fsurg.2025.1594731

**Published:** 2025-06-02

**Authors:** Barbara Cappelletto, Rossella Rispoli, Paola Peretta, Laura Grazia Valentini, Debora Garozzo, Maurizio Fornari, Concetta Alafaci, Mathew E. Diamond

**Affiliations:** ^1^ Spine and Spinal Cord Surgery Unit, Department of Head, Neck, and Neurological Sciences, University Hospital of Udine, Udine, Italy; ^2^ Division of Pediatric Neurosurgery, Children’s Hospital (O.I.R.M.), Turin, Italy; ^3^ Department of Neurosurgery, Foundation IRCCS Neurological Institute Carlo Besta, Milan, Italy; ^4^ Mediclinic Parkview Hospital, Umm Al Suqueim Barsha South, Dubai, United Arab Emirates; ^5^ Department of Neurosurgery, Humanitas University and Research Hospital in Neurosurgery, Milan, Italy; ^6^ Neurosurgery Unit, Department of Biomedical and Dental Sciences and Morphofunctional Imaging, University of Messina, Messina, Italy; ^7^ Cognitive Neuroscience, International School for Advanced Studies (SISSA), Trieste, Italy

**Keywords:** gender parity, women in neurosurgery, gender discrimination, female underrepresentation, self-segregation, Italian survey

## Abstract

The sparse representation of women in neurosurgery, compared to other medical fields, has driven debates about causes and remedies. This study explores gender disparities through a survey of female members of the Italian Society of Neurosurgery (SINch). Comprising 49 questions, the survey focused on career trajectories, work-life balance, mentorship, and perceptions of gender-related challenges. The respondents numbered 119, with 51% from 31 to 40 years old. Personal motivation emerged as the dominant driver for choosing neurosurgery. Mentorship was identified as a critical factor, with 69% perceiving benefits from having female mentors. Over half of respondents reported experiencing gender bias during residency and in the workplace. Many reported facing discouragement from peers and professors. A substantial proportion reported difficulty reconciling family responsibilities with professional duties, with 84% attributing lack of commitment to a relationship, and 49% attributing delayed motherhood, to professional demands. Respondents also expressed dissatisfaction with their visibility in scientific societies and conferences. As a remedy to hindrances of career progression, the majority of respondents do not envision female-centered initiatives (e.g., quotas in scientific societies, “women sections” or “pink rooms” at conferences). Instead, the respondents seek recognition based on merit. Rather than being categorized by their gender, the women surveyed advocate for a fair system where all individuals work on equal footing. Discussing these findings in the context of initiatives entailing quotas and enforced diversity, we argue that identity-based programs undermine core principles. Addressing underrepresentation in neurosurgery requires solutions that promote access and recognition for contributions irrespective of gender. Scientific associations have a fundamental responsibility in combating prejudice and enforcing measures to ensure the elimination of all forms of bias within neurosurgery.

## Introduction

Although women constitute a continuously growing proportion of medical professionals (1), surgical specialties remain numerically dominated by men in most nations. Proportions of surgeons identified as female in the USA range from 6% of orthopedic surgeons to 22% of general surgeons (2). In the UK in 2020, 16.1% of consultants and 34.2% of registrars working in the 10 surgical specialties (pooled together) were female (3). Among consultants, in the same analysis, the surgical sub-specialty of Neurosurgery continues to be characterized by a significant gender disparity (8.2%) and is grouped with the specialties of Cardiothoracic Surgery (10.8%) and Trauma and Orthopedics (7.3%) in the ranking of lowest female representation (3).

The aim of the present report is to better understand the current landscape in Italy, by means of a survey taken among female neurosurgeons. From these results, we proceed to consider what policies and initiatives might be taken to guarantee that talented young female medical doctors do not perceive barriers that will inhibit them from pursuing a residency in Neurosurgery and that, post-residency, the expertise of female neurosurgeons is best deployed towards the quality of health care available to the public.

The Italian Society of Neurosurgery (Società Italiana di Neurochirurgia, SINch) constitutes the context of the present work. The involvement of SINch can be best appreciated through a historical perspective. The establishment of neurosurgery as a distinct medical specialty owes much to Harvey Cushing's (1869–1939) drive for innovation in the traditional neurological and surgical fields (4). At the 1919 meeting of the American College of Surgeons, Cushing suggested that a group of peers with common interests should meet separately to “…discuss our problems and compare results” (5). Cushing and colleagues wasted no time – by 1920, the Society of Neurological Surgeons held its inaugural meeting with Cushing, naturally, a founding member. SINch was founded on May 29, 1948, with aims much like those envisioned by Cushing in the US: to serve as a platform for neurosurgeons to exchange ideas, collaborate on research, and share best practices (6). The ultimate mission of SINch is to promote progress in neurosurgery and enhance the quality of care provided to patients across Italy (6).

We composed a survey made up of 49 questions covering topics ranging from general data to career choices, work-life balance, and engagement with scientific societies. Our intention is to achieve a more nuanced understanding of the professional and personal lives, the career paths, and current status of female members of SINch, and also to uncover trends related to more subjective experiences, motivations, and perspectives. In the sections that follow, we provide the survey's findings and conclude by considering the issues female neurosurgeons have faced, and what solutions might allow them to optimally deploy their efforts towards boosting the effectiveness of the neurosurgery profession.

## Materials and methods

### Questionnaire development

After the Executive Board of SINch assessed the project as consistent with the Society's mission, the survey was launched. SINch facilitated the collection of data by allowing the authors to access the membership of the society. The survey was conducted among female neurosurgeons within SINch. The complete survey question list is provided in the original Italian and in translation to English as Supplementary Tables S1, S2. The free-text responses are given in the original Italian and in translation to English in Supplementary Tables S3, S4.

The authors crafted 49 questions, including both multiple-choice and free-text responses, covering a wide range of themes relevant to the professional and personal lives of neurosurgeons. It must be noted that questions were of three forms: (i) 36 single-response questions, where only one response could be recorded (e.g., “Do you think that patients lack confidence in a female neurosurgeon?”), (ii) 9 multiple-response questions, where more than one response could be recorded (e.g., “Who supported your choice to become a neurosurgeon?”). On the multiple-response questions, the total number of responses could surpass the number of respondents. To facilitate interpretation, a note is included in the text whenever the discussed results derive from a multiple-response question. For both single-response questions and multiple-response questions, the result is given as the percentage of respondents selecting each of the available responses. (iii) 4 free-response questions, wherein the respondent could record text (e.g., “If you have had work experience abroad, where?”).

The questionnaire underwent thorough evaluation by the SINch Executive Board to ensure its relevance, clarity, and appropriateness for the target audience. Approval to conduct the survey was communicated by the Board to the authors at the end of April 2023.

### Target population

SINch members identified as female through the Tax ID code in their registration profile with SINch were individuated as potential participants. This yielded a body of 251 individuals, the target population for the survey. Out of the 251 SINch members contacted, 8 were unreachable by email. The 119 responders represent 49% of the successfully contacted (*n* = 243) target group.

### Survey period

The online survey was conducted over a four-month period, from May 15 to September 15, 2023, allowing sufficient time for participants to respond at their convenience.

### Voluntary and anonymous participation

To foster conditions of openness and confidentiality conducive to candid feedback, participants were assured that their involvement was voluntary and would have no impact on their career status. They were informed that their responses would remain anonymous, and informed consent was implied through the completion of the survey. This study, which focused on professional experiences, career trajectories, and perceptions of members of the Italian Society of Neurosurgery (SINch), did not require ethical approval, as it involved the collection of voluntary, non-sensitive, and anonymized data without personal health information or vulnerable populations.

In accordance with institutional and national guidelines, studies that do not involve identifiable private information or interventions are exempt from ethics committee review. Nevertheless, the survey was conducted in an ethically responsible manner, adhering to principles of confidentiality and respect for all participants.

### Data analysis

The collected responses were quantified in terms of the percent of respondents who selected each of the available choices. Free-text responses were assessed qualitatively.

## Results

We report the responses of 119 female neurosurgeons, all of whom are members of SINch. Data were collected in single-response format, meaning that percent of all choices summated to 100, or in multi-response format, meaning that percent of all choices could summate to more than 100 (see Methods). Reported data are single-response unless specified as multi-response. The findings from the survey of female neurosurgeons within the SINch community reveal several significant trends and underlying challenges.

### Demographics and subspecialization (questions 1–4)

**Figure 1:**
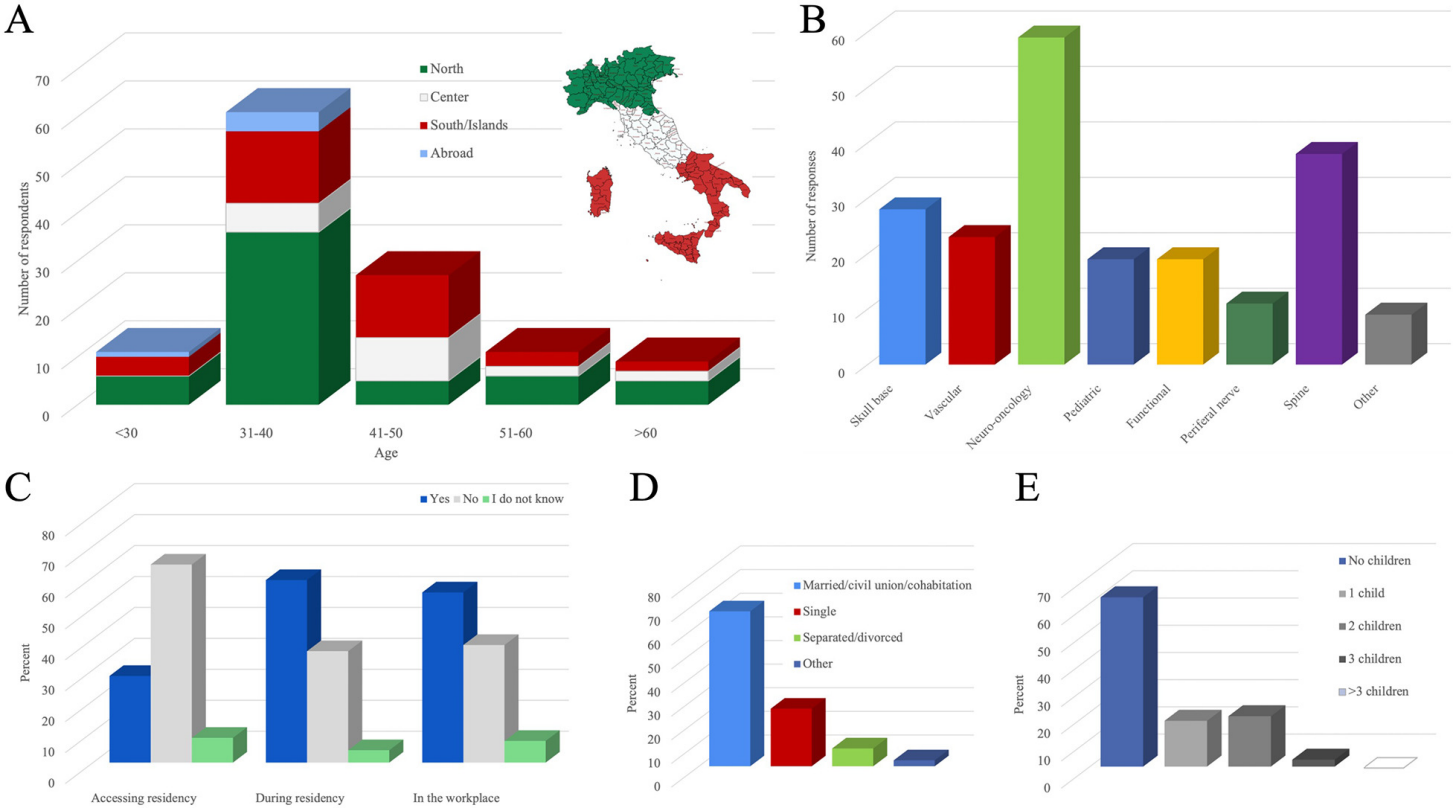
Demographic, professional, and personal profiles of surveyed neurosurgeons, with a focus on perceptions of discrimination. **(A)** Age and geographical distribution. North: Valle d'Aosta, Lombardia, Piemonte, Liguria, Trentino Alto Adige, Veneto, Friuli Venezia Giulia, Emilia Romagna. Center: Toscana, Umbria, Marche, Lazio. South/Islands: Abruzzo, Molise, Campania, Puglia, Basilicata, Calabria/Sardegna, Sicilia. **(B)** Neurosurgical sub-specializations (multiple response). **(C)** Perceived discrimination at three stages: accessing neurosurgery residency, during residency, and in the workplace. **(D)** Personal life characteristics. **(E)** Maternity.

Neurosurgeons 31–40 years old were the most prevalent respondents (51%), followed by 41–50 years (23%), over 51 years (17%), and, lastly, those <30 years (9%). This age profile was consistent across Italian regions (Figure 1A). They work primarily in public hospitals (50%) or university hospitals (37%) while the remaining 13% are employed in accredited private hospitals or other institutions.

As to subspecialization (Figure 1B), a multi-response question, neuro-oncology (50%) was most common among respondents, followed by spine surgery (32%). Less common were subspecializations in skull base, vascular, pediatric, functional, and peripheral nerve surgery.

### Motivation and influence (questions 5–7)

For 89% of respondents, there was no external influence leading to the choice to become a neurosurgeon – it was a “personal choice”. A large proportion (62%) of respondents report that the choice to practice neurosurgery was based on individual talent, while for nearly as many (55%) the choice was based on a particular interest in the field (as a multiple-response question, the percentages can summate to >100). Only 15% report that the choice was made due to inspiration by a role model. A notable percentage of respondents refer to facing discouragement (70%). Among those who feel that they were discouraged, 63% attribute the discouragement to peer-colleagues and 54% to university professors (as a multiple-response question, the percentages can summate to >100).

### Support and discrimination (questions 8–15 and 33)

Of the respondents, 89% feel they received support in their career choice. For those receiving support, 75% ascribe the support to the family, 42% to friends, and 38% to the partner. Additionally, support came from peer-colleagues (25%) and professors (20%); as support could derive from multiple sources, the percentages can summate to >100.

Discrimination was perceived during the procedure for admission to the residency by only 28% of respondents (Figure 1C). However, 59% of respondents perceived discrimination during residency. When asked who exerted the greatest degree of discrimination during residency, the offending party was defined as tenured colleagues (39%) and, secondarily, as patients (22%).

What did the perceived unfair treatment during residency consist of? Discrimination was manifested as the respondent being delegated to administrative and non-surgical tasks (57%), having limited access to learning opportunities (47%), verbal communication (44%), and being allowed less involvement in scientific activities (34%); as a multiple-response question, the percentages can summate to >100.

As seen in Figure 1C, the proportion of respondents experiencing gender discrimination increased at the transition into residency (28–59%) and remained stable (55%) as the neurosurgeon entered the workplace post-residency.

The post-residency workplace discrimination was manifested as the respondent being delegated to administrative and non-surgical tasks (57%), having limited access to learning opportunities (51%), verbal communication (36%), heavier on-call duties or other forms of discrimination (36%), and being allowed less involvement in scientific activities (27%). As a multiple-response question, the percentages can summate to >100%. The Director of the Department was considered not to have engaged in gender discrimination by 60% of the respondents. The majority of respondents (71%) think that patients lack confidence in a female neurosurgeon.

### Mentorship and international experience (questions 16 and 40–41)

Having a female mentor was deemed advantageous by 69% of respondents. Nearly half (48%) of respondents have had international experience (the list of locations is available in the Supplementary Tables S3, S4). Just under half report less (47%) or no difference (44%) in discriminatory attitudes in non-Italian settings as compared to Italian. This result implies that the Italian environment may be more gender-biased than what was found outside Italy.

### Partner relationships (questions 17 and 32)

Most respondents (68%) are in stable personal relationships (marriage, civil union, cohabitation), indicating some priority given to personal commitment (Figure 1D). Of the remaining 32% of respondents (single, separated, or divorced), a clear majority (84%) attributed, completely or partially, their lack of current commitment to a relationship to the demands of neurosurgery.

### Challenges of motherhood and career (questions 18–27 and 30–31)

More than half (62%) reported having no children (Figure 1E). Of those who decided not to have children (34% of all respondents), this decision depended partially or to a small degree (78%) or else completely (12%) on the fear of not being able to balance career and family. Thus, 31% of all respondents (90% of 34%) renounced motherhood in order to prioritize neurosurgery. Only 10% would not have wanted children, independently of work demands.

About half (49%) reported postponing motherhood due to work issues, and 19% reported having problems becoming pregnant. Miscarriage was reported by 13%.

Almost half (44%) of the respondents with children reduced their maternity leave to avoid problems at work (duration of maternity leave is listed in the Supplementary Tables S3, S4). The spouse/partner's option for paternity leave was utilized by only 7% of couples.

A significant percentage 64% believe that motherhood imposed career limitations, and 40% believe that the department chief limited professional growth opportunities upon returning from maternity leave. After maternity leave 44% sacrificed family life for work while 28% made sacrifices at work to give time to the family. On the other hand, 28% did not report sacrificing either work or family. A sense of guilt or inadequacy for not being able to give enough attention or time to their children due to work commitments was reported by 49% of mothers.

### Support systems and renunciation of opportunities after motherhood (questions 28 and 29)

Respondents with children identified their own parents (76%) and partner/husband (76%) as the most helpful in organizing family life, with contribution from nurseries (44%) and friends (24%). A considerable percentage of respondents reported giving up training/courses/congresses (58%), outpatient work/private practice (26%), operating room duties (26%), and research/university careers (23%) after having children. As both were multiple-response questions, the percentages can summate to >100.

### Access to top positions, career advancement, and conference visibility (questions 34–36)

**Figure 2:**
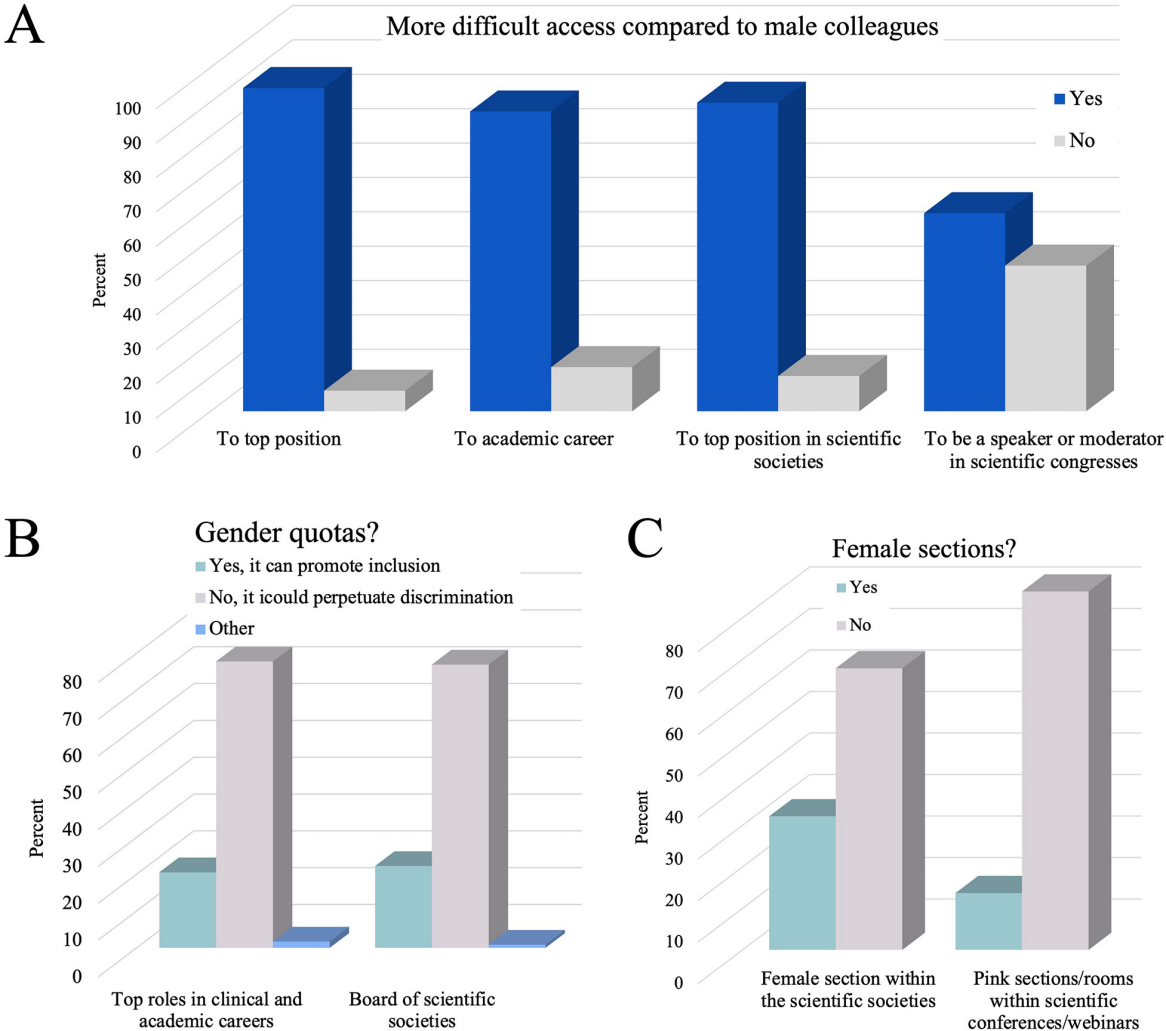
Perspectives on gender disparities and representation in neurosurgery. **(A)** Respondents’ experiences with barriers to accessing top hospital positions, academic careers, leadership roles in scientific societies, and opportunities as speakers or moderators at scientific congresses. **(B)** Disagreement among respondents regarding the introduction of gender quotas in clinical and academic careers and scientific society boards. **(C)** The respondents’ opposition to the creation of female-only sections within the scientific societies or dedicated “pink” spaces within scientific conferences.

An overwhelming majority of respondents (94%) reported experiencing difficulties in accessing top positions. Similarly, achieving a university career (87%) and securing roles as moderators or speakers at conferences (58%) were also perceived as challenging for female neurosurgeons (Figure 2A).

### Representation in scientific societies, views on quotas and gender-specific initiatives (questions 37–39 and 43–47)

A vast majority of respondents (90%) reported experiencing difficulties in accessing top positions within scientific societies (Figure 2A). Around half (48%) would be interested in being part of the board of a scientific society. A slight majority (56%) of respondents do not feel adequately represented in scientific societies.

Despite the challenges faced, most respondents (78%) are not in favor of the introduction of female quotas in top roles in clinical and academic career (Figure 2B). Likewise, most respondents (77%) are not in favor of female quotas in the boards of the scientific societies (Figure 2B).

Additionally, most respondents (68%) are against the introduction of “women's sections” within scientific societies and a substantial majority of respondents (86%) are against “pink rooms” at conferences (Figure 2C). The majority (79%) expressed the need to establish a task force within scientific societies to combat all forms of discrimination.

### Promotion of work-family conciliation (question 42)

What can be done to facilitate the full participation of female neurosurgeons? A high proportion of respondents advocate for measures to promote work-family conciliation, such as the establishment of nurseries within workplaces (89%), the de-taxation of domestic collaboration contracts for healthcare workers (58%), encouraging the use of paternity leave (50%), and “regulated placements” in daycare/nursery schools for healthcare professionals (40%). As a multiple-response question, the percentages can summate to >100.

### Descriptive questions (48 and 49)

The texts describing episodes of discrimination experienced and the suggestions for improving the future path are reported in Supplementary Tables S3, S4.

## Discussion

### Motivation and commitment

The survey results highlight that women entering neurosurgery are driven by profound intrinsic motivation and a deep passion for the field. An overwhelming 89% of respondents indicated that their decision to pursue neurosurgery was rooted in personal aptitude and interest, not external pressures. This underscores that women in neurosurgery are not seeking validation through conformity to societal or professional expectations but are instead committed to excelling in a challenging and rewarding discipline.

Notwithstanding the pressures of the career, 68% of respondents were in stable relationships, suggesting that personal commitments can coexist with a demanding career in neurosurgery. Conversely, 84% of those who were single, separated, or divorced attributed their relationship status to the pressures of their profession, highlighting the toll it can take on personal lives.

Nearly half (49%) of respondents reported delaying motherhood due to work-related reasons, and 31% chose to forego motherhood entirely to prioritize their careers. These figures reflect the significant personal sacrifices women make to advance in neurosurgery. The survey also revealed that 64% of mothers experienced professional limitations after having children, with many (44%) sacrificing family life and others (28%) making professional sacrifices upon returning from maternity leave. These findings illustrate the difficult choices women in neurosurgery must navigate to balance professional and personal responsibilities. Nearly half of the respondents with children reported feelings of guilt or inadequacy in attempting to balance these dual roles.

In light of these challenges, respondents strongly advocated for measures to promote work-family balance. Popular suggestions included the establishment of workplace nurseries, tax benefits for domestic assistance, and guaranteed access to daycare facilities for healthcare professionals. Encouraging the use of paternity leave was also widely supported. The analysis was based on the responses of 119 neurosurgeons, representing 49% of those contacted. It is unclear how the trends uncovered might be affected by this incomplete sampling. Those neurosurgeons who chose not to respond, or who have left the profession, might be those facing even greater hardship, but this speculation cannot be verified.

The results of the present survey bear some resemblance to a recent survey which included the data of Italian female neurosurgeons by Scerrati et al. (7). The key differences between the present study and that of (7) concern both the target population and the survey's objectives. The previous work extrapolated insights about female neurosurgeons from a broader cohort of women surgeons across various specialties. In contrast, our survey was specifically designed for neurosurgeons and was conducted through the Italian Neurosurgical Society (SINch), allowing us to collect a focused and representative dataset tailored to this specialty. Our questionnaire included detailed items on neurosurgical subspecialties to better capture the distribution of interests and professional roles among female SINch members. In addition, we investigated themes related to career progression and the promotion of work-family balance. Crucially, we incorporated two open-ended questions inviting respondents to share personal experiences of discrimination and to suggest strategies for fostering greater involvement in the field. Perhaps the most salient distinction lies in the broader scope and intent of our survey. While (7) provided a snapshot in time, our study offers a more dynamic perspective on the ongoing pursuit of equal opportunity in neurosurgery.

### Limitations of the study

One technical limitation of the survey design was the lack of question gating, which resulted in minor inconsistencies in response numbers for related questions. For instance, 44 participants answered the question, “If you are single/separated/divorced, do you believe that being a neurosurgeon has affected your personal relationships?” Responses to this question were calculated only for the 38 participants who had previously indicated being single, separated, or divorced. This discrepancy suggests that a small number of participants chose to highlight relationship issues even though they had not explicitly identified with the relevant status. It may also be criticized that participants' assessments of their careers, both in the multiple-choice section and the free response section, were subjective. This is because our intention is to document the perceived experience through the eyes of the participants as much as the documentable, objective career markers.

### Interpreting the findings through the lens of history

The challenges faced by women in neurosurgery are best understood in relation to principles that emerged during the 17th and 18th century Enlightenment, a period when the mobility of individuals within society began to be conceived of as being rooted in their abilities and contributions rather than by immutable characteristics such as inherited class, gender, race, or religion (8–12). Such revolutionary ideals challenged the hierarchical norms of earlier eras and laid the groundwork for modern concepts of inalienable rights and equality under law.

However, a contemporary shift towards identity-based frames of reference (13) threatens to undermine these principles. When individuals are awarded (or denied) opportunity and recognition based on labels—such as gender—rather than their abilities, institutions risk returning to a pre-Enlightenment state where one's identity determines one's fate. The respondents in the present survey appear to reject this option. While many feel held back in certain aspects of their careers, the restricted opportunities are not perceived as an insurmountable barrier but rather as a motivation to push for equal access and visibility. They do not want to be seen as representatives of their gender but as individuals with unique talents and contributions to offer. The refutation of quotas and other forms of enforced diversity can thus be understood as a push for recognition of individual merit and responsibility. As such, any move toward contrived diversity risks reversing roles in ways that hurt everyone, reinforcing a cycle of division rather than integration. The strategy *least* likely to open the doors of opportunity is active training (lectures, exams, seminars) ostensibly designed to educate participants about prejudice and bias: this pedagogical approach is ineffective (14) and even appears to, paradoxically, augment prejudicial attitudes among trainees (15).

Consistent with the conclusion of the present work, a previous paper (16) argued that organizations like Women in Neurosurgery (WINS, founded 1989) contributed at their outset to promoting a culture of opportunity for females, thus playing a pivotal role in combatting prejudice. However, the existence of WINS is by now anachronistic and should no longer be necessary in an era of authentic equal opportunity (16). We suggest that associations of women in neurosurgery (or any other professional field) are counterproductive, since they may act to perpetuate rather than dissolve divisions. This has been seen historically in the formation of racially exclusive organizations. For example, in the United States the Black-only National Medical Association arose from the exclusion of African-American doctors from mainstream medical associations, but in later years it further entrenched divisions in the medical community (17). Attempts to amalgamate both organizations have routinely failed over the years (18). Self-dissociation of marginalized groups, even if justified at the time of its enactment, often leads in the long term not to unity but to bifurcation. Our survey supports this lesson – respondents did not indicate any intent to defend their rights by dissociating themselves from male colleagues.

### Grounds for optimism

While we acknowledge that 69% of respondents in the current study responded favorably to the possibility of having a female mentor, we can posit that free and fair chances for women neurosurgeons will bring to bear a much larger pool of female neurosurgeons within a few years, ready to act as mentors. Women have already achieved significant representation in many other medical specialties, including branches of surgery outside of neurosurgery. The progress observed in these fields demonstrates that barriers to full participation can be overcome when opportunities are distributed fairly. While neurosurgery has lagged behind in achieving gender parity, the data suggest that with continued effort to ensure fair access and equitable evaluation, women can thrive and make substantial contributions to the field.

Interestingly, many respondents reported experiencing less gender-based discrimination while working outside of Italy. This suggests that international practices and policies could serve as valuable models for reducing bias within Italian neurosurgery.

Addressing the challenges faced by women in neurosurgery requires a commitment to professionalism and fairness. This involves not only eliminating discrimination but also ensuring that opportunities for growth and advancement are available to all based on individual merit. By fostering an environment that values contributions over identities, neurosurgery can continue to attract and retain the best talent, ensuring the continued evolution and excellence of the field.

In summary, while the gender disparity in neurosurgery persists (19), the societies like SINch and the European Association of Neurosurgical Societies (EANS) can play a pivotal role in the fight against prejudice and can promote the culture of change. The boards of each society should oversee and enforce measures that promote fair opportunities. The EANS is already moving in this direction by establishing a task force focused on broader diversity issues (20). The continued development of neurosurgery can be achieved only through promotion of professionalism, regardless of the gender of those who practice this challenging surgical specialty.

## Data Availability

The raw data supporting the conclusions of this article will be made available, upon reasonable request to the corresponding author.
