# Supplementary material for: Women in neurosurgery aim for recognition of merit, not tokenism: insights from an Italian survey
Source: Front Surg. 2025 Jun 2;12:1594731. doi: 10.3389/fsurg.2025.1594731 (PMC12171119; doi:10.3389/fsurg.2025.1594731)
Supplement: Supplementary file 1 [file Table1.docx]

Questionario

| **Dati Generali** |
| --- |

1. Età

- <30
- 30-40
- 40-50
- 50-60
- > 60

1. Istituzione dove si svolge la professione

- Ospedale Pubblico
- Ospedale Privato Convenzionato
- Ospedale Universitario
- Altro

1. Luogo dove si svolge la professione

- Nord
- Centro
- Sud / Isole
- Estero

1. Sub-specializzazione neurochirurgica (scelta multipla)

- Basicranio
- Vascolare
- Oncologica
- Pediatrica
- Funzionale
- Chirurgia del periferico
- Chirurgia spinale
- Altro

| **Gli Inizi: Motivazionale** |
| --- |

1. La tua scelta di diventare neurochirurgo è stata influenzata da altri?

- No, è stata una scelta personale
- Sono stata consigliata da altra persona
- Sono stata scoraggiata
- Altro

1. Perché la Neurochirurgia? (scelta multipla)

- Per mettere a frutto delle attitudini personali
- Per affinità con la materia
- Per il rapporto coi pazienti
- Perché ispirata da qualcuno
- Altro

1. Chi ha cercato di convincerti che non era una scelta opportuna? (scelta multipla)

- La mia famiglia
- Partner di allora
- Gli amici
- I professori all’università
- I colleghi
- Nessuno

1. Chi ti ha supportato nella scelta? (scelta multipla)

- La mia famiglia
- Partner di allora
- Gli amici
- I professori all’università
- I colleghi
- Nessuno

| **La Realtà 1: Studi e Vita Professionale** |
| --- |

1. Hai sperimentato discriminazioni in quanto donna nell’accesso alla scuola di specializzazione in neurochirurgia?

- Si
- No
- Non so

1. Pensi di essere stata discriminata durante il percorso di specializzazione?

- Si
- No
- Non so

1. Chi ti ha fatto oggetto di discriminazione di genere durante la specializzazione?

- I colleghi specializzandi
- I colleghi strutturati
- I docenti
- Il personale
- I pazienti
- Nessuno

1. Che tipo di discriminazione hai subito durante la specializzazione? (scelta multipla)

- Verbale
- Limitazioni nell’accesso all’apprendimento
- Delegata più frequentemente ad incarichi amministrativi e non-chirurgici
- Minor coinvolgimento nelle attività scientifiche
- Nessuna

1. Sul posto di lavoro (sia in passato che dove lavori attualmente), reputi di essere stata o essere oggetto di discriminazione di genere?

- Si
- No
- Non so

1. Che tipo di discriminazione hai subito o subisci sul posto di lavoro? (scelta multipla)

- Verbale
- Limitazioni nell’accesso alla sala operatoria rispetto ai colleghi maschi
- Delegata più frequentemente ad incarichi amministrativi e non-chirurgici
- Minor coinvolgimento nelle attività scientifiche
- Carico di guardie più gravoso
- Nessuna
- Altro

1. Reputi che il tuo direttore faccia discriminazioni di genere?

- Si
- No
- Non so

1. Se ha avuto occasione, reputi che sia un’esperienza positiva lavorare con una donna neurochirurgo di maggiore esperienza?

- Si
- No
- Non so

| **La Realtà 2: Lavoro & Vita Personale** |
| --- |

1. Vita personale

- Matrimonio/unione civile/convivenza
- Single
- Separata/divorziata
- Altro

1. Maternità

- No figli
- 1 figlio
- 2 figli
- 3 figli
- > 3 figli

**Se sei una donna con figli, compila anche le seguenti 19-30:**

1. Hai scelto di posticipare la maternità per problemi di lavoro?

- Si
- No

1. Hai avuto problemi a rimanere incinta?

- Si
- No

1. Hai avuto aborti spontanei?

- Si
- No

1. Quanto tempo sei stata assente dal lavoro per le maternità (in totale)?

…….. mesi

1. Hai ridotto il periodo di maternità (sia per la maternità biologica che in caso di adozione) per evitare problemi sul lavoro?

- Si
- No

1. Il tuo marito/compagno ha preso un periodo di congedo per la maternità

- Si
- No

1. Reputi che la/e maternità abbia/no comportato delle limitazioni alla tua carriera?

- Si
- No

1. Dopo la/le maternità, il tuo primario ti ha dato le stesse possibilità di crescita professionale che avevi prima o che avevano gli altri colleghi?

- Si
- No

1. Dopo la/le maternità hai deciso tu di ridurre il tuo impegno lavorativo?

- Si, ho dovuto fare delle rinunce sul lavoro per conciliare famiglia e lavoro
- No, ho scelto di fare delle rinunce sulla famiglia per continuare a lavorare
- Non applicabile
- Altro

1. Persone/istituzioni che ti hanno supportato: (scelta multipla)

- I miei genitori
- Il mio compagno/marito
- Amiche
- Nidi d’infanzia
- Altro

1. Se hai dovuto fare delle rinunce lavorative, a cosa hai rinunciato: (scelta multipla)

- alla ricerca/carriera universitaria
- alla formazione/corsi/congressi
- alla sala operatoria
- all’attività ambulatoriale/libera professione
- al Pronto Soccorso
- altro

1. Se hai figli, provi un senso di colpa o inadeguatezza per non riuscire a dedicare ai tuoi figli le attenzioni o il tempo che vorresti a causa del tuo lavoro?

- Si
- No

1. Se hai scelto di non avere figli, quanto è dipeso dal timore di non poter conciliare carriera e famiglia?

- Per nulla, non avrei voluto avere figli comunque
- Poco
- In parte
- Del tutto dal lavoro

1. Se sei single/separata/divorziata, ritieni che essere neurochirurgo abbia influito sulla tua vita affettiva?

- Per nulla, non avrei voluto una vita di coppia comunque
- Poco
- In parte
- Del tutto dal lavoro
- Non applicabile

1. Reputi che da parte dei pazienti ci sia diffidenza verso un neurochirurgo donna?

- Si
- No
- In parte
- Lo era in passato, oggi non più o solo eccezionalmente

1. Reputi che in Italia per una donna neurochirurgo accedere a posizioni apicali sia più difficile che per un uomo?

- Si
- No

1. Ritieni che in Italia per una donna neurochirurgo sia più difficile fare carriera universitaria?

- Si
- No

1. Ritieni che per una donna neurochirurgo sia più difficile essere moderatore o speaker nelle sessioni congressuali?

- Si
- No

1. Reputi che sia più difficile per una donna neurochirurgo accedere a posizioni apicali all’interno delle società scientifiche?

- Si
- No

1. Sei mai stata interessata di far parte del comitato direttivo di una società scientifica?

- Sì e l’ho fatto
- Sì, ma per ora non ho ancora provato a farlo
- Sì, ma ho dovuto rinunciare perché impossibile
- No

1. Ti senti rappresentata all’interno delle società scientifiche italiane/ internazionali?

- Sì
- No
- Altro

1. Se hai avuto un’esperienza di lavoro all’estero, dove?

Nazione/i:

1. Se hai avuto un’esperienza di lavoro all’estero, ti sei sentita discriminata rispetto all’Italia?

- Di più
- Di meno
- Non ho rilevato differenze
- Altro

| **Il Futuro: Correttivi possibili** |
| --- |

1. Per promuovere la conciliazione lavoro/famiglia, quali di questi strumenti vorresti che venissero supportati? (scelta multipla)

- Congedi di paternità (come avviene all’estero)
- Promozione di nidi/scuole materne all’interno dei luoghi di lavoro
- De-tassazione dei contratti di collaborazione domestica per i sanitari
- Accesso a liste con “quote calmierate” nei nidi-scuole materne per i sanitari
- Altro

1. Sei favorevole all’introduzione delle “quote rosa” nei ruoli apicali nella carriera clinica ed accademica, come avviene per i ruoli dirigenziali in alcune società private?

- Si, può favorire l’inserimento delle donne neurochirurgo
- No, è un ulteriore modo (sia pure indiretto) di perpetrare la discriminazione delle donne neurochirurgo; siamo tutti professionisti indipendentemente dal genere e la selezione deve essere basata solo sulla qualità professionale
- Altro

1. Sei favorevole all’introduzione delle “quote rosa” nelle società scientifiche, come ha suggerito qualcuno?

- Si, può favorire l’inserimento delle donne neurochirurgo
- No, è un ulteriore modo (sia pure indiretto) di perpetrare la discriminazione delle donne neurochirurgo; siamo tutti professionisti indipendentemente dal genere e la selezione deve essere basata solo sulla qualità professionale
- Altro

1. Ritieni opportuna la creazione di una sezione “Donne in Neurochirurgia” (sul modello della WINS “Women in NeuroSurgery” della WFNS) all’interno delle società scientifiche?

- Si
- No

1. Credi che sia utile per promuovere la partecipazione delle donne nelle società scientifiche organizzare sezioni “rosa” all’interno delle conferenze scientifiche, webinar ecc. in cui parlino solo donne neurochirurgo?

- Si, può essere un modo valido per evidenziare e valorizzare il lavoro delle donne neurochirurgo, favorendone il networking e la solidarietà
- No, risulterebbe in una forma di autoemarginazione che rischia di dividere la comunità neurochirurgica sulla base del genere invece di promuovere l’uguaglianza e la professionalità super partes
- Altro

1. Sei favorevole alla creazione di una “task force” all’interno delle società scientifiche per combattere ogni forma di discriminazione (di genere, razziale, religiosa ecc.) sul modello di quanto esistente nell’EANS, ISPN ecc..?

- Si
- No, non ne sento la necessità
- Altro

1. Se ti è capitato, descrivi brevemente un episodio di discriminazione che hai subito:

……………………………………………………………………………………………………………………………………

……………………………………………………………………………………………………………………………………

1. Hai proposte per migliorare il percorso futuro:

……………..……………………………………………………………………………………………………………………

……………………………………………………………………………………………………………………………………
