# Supplementary material for: Women in neurosurgery aim for recognition of merit, not tokenism: insights from an Italian survey
Source: Front Surg. 2025 Jun 2;12:1594731. doi: 10.3389/fsurg.2025.1594731 (PMC12171119; doi:10.3389/fsurg.2025.1594731)
Supplement: Supplementary file 2 [file Table2.docx]

Questionnaire

| **General Data** |
| --- |

1. Age

- <30
- 30-40
- 40-50
- 50-60
- > 60

1. Institution where you practice

- Public Hospital
- Accredited Private Hospital
- University Hospital
- Other

1. Territory of practice

- North
- Center
- South / Islands
- Abroad

*North: Valle d’Aosta, Lombardia, Piemonte, Liguria, Trentino Alto Adige, Veneto, Friuli Venezia Giulia, Emilia Romagna

Center: Toscana, Umbria, Marche, Lazio

South / Islands: Abruzzo, Molise, Campania, Puglia, Basilicata, Calabria / Sardegna, Sicilia

1. Neurosurgical sub-specialization (multiple response)

- Skull base
- Vascular
- Neuro-oncology
- Pediatric
- Functional
- Peripheral nerve
- Spinal
- Other

| **The beginnings: Motivation** |
| --- |

1. Was your choice to become a neurosurgeon influenced by others?

- No, it was a personal choice
- I was advised by someone else
- I was discouraged
- Other

1. Why Neurosurgery? (multiple response)

- To apply my individual talents
- Because of an interest in the field
- For the relationship with patients
- Because inspired by a role model
- Other

1. Who tried to convince you that it was not a suitable choice? (multiple response)

- My family
- Partner at that time
- Friends
- Professors at university
- Colleagues
- Nobody

1. Who supported you in your choice? (multiple response)

- My family
- Partner at that time
- Friends
- Professors at university
- Colleagues
- Nobody

| **Reality 1: Studies and Professional Life** |
| --- |

1. Did you experience discrimination as a woman in accessing the neurosurgery residency program?

- Yes
- No
- I do not know

1. Do you think you were discriminated against during your residency?

- Yes
- No
- I do not know

1. Who subjected you to gender discrimination during your residency?

- Fellow residents
- Tenured colleagues
- Teachers/tutors
- Staff
- Patients
- Nobody

1. What type of discrimination did you experience during your residency? (multiple response)

- Verbal
- Limitations in access to learning
- More frequently delegated to administrative and non-surgical tasks
- Less involvement in scientific activities
- None

1. In the workplace (both in the past and at your current job), do you believe you have been or are currently a victim of gender discrimination?

- Yes
- No
- I do not know

1. What type of discrimination have you experienced or are you experiencing in the workplace? (multiple response)

- Verbal
- Limitations in access to the operating room compared to male colleagues
- More frequently delegated to administrative and non-surgical tasks
- Less involvement in scientific activities
- Heavier on-call duties
- None
- Other

1. Do you believe that your director engages in gender discrimination?

- Yes
- No
- I do not know

1. If you have had the opportunity, do you consider it a positive experience to work with a more experienced female neurosurgeon?

- Yes
- No
- I do not know

| **Reality 2: Work & Personal Life** |
| --- |

1. Personal life

- Marriage/civil union/cohabitation
- Single
- Separated/divorced
- Other

1. Maternity

- No children
- 1 child
- 2 children
- 3 children
- > 3 children

**If you are a woman with children, please fill out the following questions 19-30:**

1. Have you chosen to postpone motherhood due to work issues?

- Yes
- No

1. Have you had trouble getting pregnant?

- Yes
- No

1. Have you experienced miscarriages?

- Si
- No

1. How much time have you been absent from work for maternity leave (in total)?

…….. months

1. Have you shortened your maternity leave (both for biological maternity and in the case of adoption) to avoid work-related issues?

- Yes
- No

1. Has your husband/partner taken paternity leave?

- Yes
- No

1. Do you believe that motherhood has imposed limitations on your career?

- Yes
- No

1. After your maternity leave, did your supervisor provide you with the same opportunities for professional growth that you had before or that other colleagues had?

- Yes
- No

1. After your maternity leave(s), did you decide to reduce your work commitment?

- Yes, I had to make sacrifices at work to balance family and work
- No, I chose to make sacrifices in my family life to continue working
- Not applicable
- Other

1. People/institutions that have supported you (multiple response)

- My parents
- My partner/husband
- Friends
- Daycare centers
- Other

1. If you had to make work-related sacrifices, what did you give up? (multiple response)

- Academic research/career
- Training/courses/conferences
- Time in the operating room
- Outpatient work/private practice
- Emergency room
- Other

1. If you have children, do you feel a sense of guilt or inadequacy for not being able to give your children the attention or time you would like because of your work?

- Yes
- No

1. If you have chosen not to have children, how much did this decision depend on the fear of not being able to balance career and family?

- Not at all, I wouldn't have wanted to have children anyway
- A little
- Partially
- Completely due to work
- Not applicable

1. If you are single/separated/divorced, do you believe that being a neurosurgeon has affected your personal relationship?

- Not at all, I wouldn't have wanted a couple's life anyway
- A little
- Partially
- Completely due to work
- Not applicable

1. Do you think that patients lack confidence in a female neurosurgeon?

- Yes
- No
- Partially
- It was in the past, but not anymore or only exceptionally

1. Do you believe that in Italy it is more difficult for a female neurosurgeon to access top positions compared to a male?

- Yes
- No

1. Do you believe that in Italy it is more difficult for a female neurosurgeon to advance in an academic career?

- Yes
- No

1. Do you think it is more difficult for a female neurosurgeon to be a moderator or speaker at congress sessions?

- Yes
- No

1. Do you believe that it is more difficult for a female neurosurgeon to access top positions within scientific societies?

- Yes
- No

1. Have you ever been interested in being part of the board of a scientific society?

- Yes, and I did it
- Yes, but I haven't tried to do it yet
- Yes, but I had to give it up because it was impossible
- No

1. Do you feel represented within Italian/international scientific societies?

- Yes
- No
- Other

1. If you have had work experience abroad, where?

Country:

1. If you have had work experience abroad, did you feel discriminated against compared to Italy?

- More
- Less
- I did not notice any differences
- Other

| **The Future: Possible Corrections** |
| --- |

1. To promote work/family balance, which of these measures would you like to see supported? (multiple response)

- Paternity leave (as it happens abroad)
- Promotion of daycare/nursery schools within workplaces
- Tax exemption for domestic collaboration contracts for healthcare professionals
- Access to lists with “regulated placements” in daycare/nursery schools for healthcare professionals
- Other

1. Are you in favor of introducing “gender quotas” in top roles in clinical and academic careers, as is done for managerial roles in some private companies?

- Yes, it can promote the inclusion of female neurosurgeons
- No, it is another way (albeit indirect) to perpetuate the discrimination against female neurosurgeons; we are all professionals regardless of gender, and selection should be based solely on professional quality
- Other

1. Are you in favor of introducing “gender quotas” in the scientific societies, as some have suggested?

- Yes, it can promote the inclusion of female neurosurgeons
- No, it is another way (albeit indirect) to perpetuate the discrimination against female neurosurgeons; we are all professionals regardless of gender, and selection should be based solely on professional quality
- Other

1. Do you think it is appropriate to create a “Women in Neurosurgery” section (modeled after WINS “Women in NeuroSurgery” of WFNS) within scientific societies?

- Yes
- No

1. Do you think it is useful to promote the participation of women in scientific societies by organizing “pink” sections within scientific conferences, webinars, etc., where only female neurosurgeons speak?

- Yes, it can be a valid way to highlight and value the work of female neurosurgeons, promoting networking and solidarity
- No, it would result in a form of self-segregation that risks dividing the neurosurgical community based on gender instead of promoting equality and professionalism beyond gender
- Other

1. Are you in favor of creating a “task force” within scientific societies to combat all forms of discrimination (gender, racial, religious, etc.), similar to what exists in EANS, ISPN, etc.?

- Yes
- No, I don’t see the need for it
- Other

1. If it has happened to you, briefly describe an episode of discrimination you have experienced:

…………………………………………………………………………………………………………………………………………

……………………………………………………………………………………………………………………………………

1. Do you have any suggestions for improving the future path?

…………………………………………………………………………………………………………………………………………

……………………………………………………………………………………………………………………………………
