# Supplementary material for: Women in neurosurgery aim for recognition of merit, not tokenism: insights from an Italian survey
Source: Front Surg. 2025 Jun 2;12:1594731. doi: 10.3389/fsurg.2025.1594731 (PMC12171119; doi:10.3389/fsurg.2025.1594731)
Supplement: Supplementary file 3 [file Table3.docx]

Domanda 22

**Quanto tempo sei stata assente dal lavoro per le maternità (in totale)?**

# 45 risposte

1 anno

10 mesi

10 mesi

# 10 mesi, per entrambi i figli, comprese le ferie

11 mesi

12 mesi

12 mesi

12 MESI

12 mesi

14 mesi

14 mesi

14 mesi

16 mesi

18 mesi

18 mesi

2 anni

2 anni per le 2 gravidanze

22 mesi

24 mesi di 2 maternità ( una peraltro ancora in atto, ho conteggiato i mesi in cui sarò ancora assente)

3 anni

3 anni

3 mesi

4 mesi

5 mesi

5 mesi

5 mesi

5 mesi

5 mesi primo figlio
5 mesi gemelli

6 mesi

6 mesi

6 mesi

6 mesi per ogni figlia

6 mesi primi figlio
5 mesi secondo figlio

7 mesi

8 mesi

9 mesi

meno di un anno in specializzazione

Pochi mesi

Sei mesi

Sei mesi

Tre mesi

Un anno

Un anno

Un mese

# In corso

**Domanda 40**

**Se hai avuto un’esperienza di lavoro all’estero, dove?**

57 hanno avuto esperienza all’estero nelle sedi sotto riportate

Cambridge

Est Europa

Fellowship New York

Francia

Francia

Francia

Francia

Francia

Francia

Francia

Francia Svizzera Germania

Francia un anno tra il 2000 e il 2001

Francia, Lione

Germania

Ginevra

Ginevra, Svizzera

Hannover- Germania

Hannover, Germania

Helsinki

Inghilterra

Leeds, uk

Lione

Lione, Londra, San Francisco, ma per periodi brevi

Londra

Los Angeles

Malaga

Mayo Clinic Rochester, istanbul, Messico, Germania

Messico

Montpellier

new york

Nord Europa

NYC

Oxford, UK

Parigi (1 anno specializzazione) e Cincinnati (7 mesi ricerca su cadavere)

Parigi, Orlando, Alicante

San Francisco

Stati Uniti

Stati Uniti

Stati Uniti

Stati Uniti, California

Strasburgo

Studio Inghilterra e California

Svezia

Svizzera

Svizzera

Svizzera

Svizzera

Svizzera e Francia

Tolosa

UK

USA

USA

USA

USA

USA

Varie

Zurigo

**Domanda 48**

**Se ti è capitato, descrivi brevemente un episodio di discriminazione che hai subito:**

# 37 risposte

| Frase di un primario all’assunzione: hai 38 anni sei vecchia e ringrazia per il posto (dopo 24 concorsi arrivata seconda o prima in 8 anni) Però potresti ancora rimanere incinta e quindi non investo su di te.. Tante altre -Dopo un concorso: sei brava ma il tuo collega ha tre figli e mi dispiace ma devo assumere lui poveretto ( io.. magari ne avessi potuti avere anch’io)… - sei troppo giovane.. - sei troppo vecchia - parli troppo ( quando in realtà gli uomini parlano sempre) Etc etc |
| --- |
| "Se non avrai figli almeno per i prossimi 10 anni, potresti diventare un bravo chirurgo" |
| all'esame di specialità il professore mi ha chiesto come mai da donna sceglievo la neurochirurgia visto che è incomaptibile con una famiglia.  ancora oggi I PAZIENTI mi chiedono di presentargli un bravo chirurgo |
| alla seconda gravidanza non ho avuto rinnovo contratto e ho fatto causa con il comitato pari opportunità |
| Allontanamento dal campo operatorio |
| Dopo 5 anni da specialista non posso fare un ernia discale da sola quando un neo specialista fa lo spinale semplice in autonomia. |
| Durante i primi anni di specialità essere sempre sfavorita rispetto ai colleghi uomini in quanto donna |
| Durante un colloquio mi è stato chiesto se avevo prospettive di una gravidanza |
| Durante uno dei periodi di formazione in ospedale all'interno della rete formativa, ad un mio collega uomo è stata data la possibilità di partecipare a più di un lavoro scientifico. A me, che pure ho espresso il desiderio di partecipare alle attività di ricerca, è stato dato da fare soltanto un lavoro, alla fine del periodo, e per di più di taglio infermieristico. |
| È abbastanza frequente la diffidenza dei/delle pazienti riguardo alle mie conclusioni diagnostiche e terapeutiche soprattutto durante le visite ambulatoriali. Spesso fanno i confronto con quanto proposto da un collega maschio fidandosi di più delle loro proposte che delle mie. |
| Essere chiamata infermiera, rimanere fuori dalla sala perché non considerata all’altezza o troppo debole per il tipo di chirurgia |
| Essere sempre chiamata “dottoressina” da uno strutturato. Sempre con lo stesso strutturato nella stanza di un paziente, io con scrub e camice, lui in borghese,lui è stato identificato dal paziente come dottore, io invece sono stata chiamata signorina. La reazione dello strutturato è stata una fragorosa risata. Sono gli episodi che mi vengono in mente subito, ma ce ne sono stati sicuramente molti altri. Non ho tuttavia mai subito limitazioni in sala operatoria, nè per quanto riguarda le attività formative in generale. Encomio al mio primario che ha in equipe 4 donne e che non fai nessuna discriminazione! |
| Gli episodi sono troppi |
| Ho colleghi maschi che possono assentarsi per i figli mentre se lo fa una donna viene criticata e esclusa. Nella mia vita professionale ho dovuto dare sempre più dei miei colleghi maschi per ottenere meno. |
| Ho dovuto in anticipo rinunciare al congedo per allattamento perche altrimenti mi era reso difficoltoso l'accesso alla sala operatoria (possibile all'interno delle ore di servizio dopo il 7 mese post parto) |
| Ho subito più intralci da una collega neurochirurga più anziana che dai colleghi maschi. Ora il rapporto fra colleghi e' assolutamente paritario. Morale: a volte possiamo essere anche nemiche di noi stesse |
| Il padre di una paziente a fine intervento invece di parlare con me che ero il chirurgo parlava solo con l’anestesista perchè era uomo |
| Il paziente ha mi ha chiesto di parlare con “il dottore” riferendosi al mio collega maschio. |
| Il primario (Prof. S.) mi ha fermato dal giro visita per farlo continuare da uno specializzando del primo anno, dicendo che io nn ho le conoscenze in quanto assentata per lungo periodo (maternità, convalescenza per patologia tumorale) |
| La maggioranza dei pazienti non mi percepisce in quanto neurochirurgo, in grado di operare. Spesso vengo scambiata per l'infermiera o l'OS. |
| Mancata possibilità di rimandare corso di formazione europeo per gravidanza ed allattamento |
| Mi è sicuramente capitato di essere stata scoraggiata ad iniziare questa carriera però una volta che ho cominciato ho trovato persone favorevoli che mi hanno incoraggiato e supportato molto |
| Mi è stato suggerito di interrompere la gravidanza per privilegiare il lavoro Sono stata oggetto di perpetuate non richieste proposte sessuali Mi è stato detto che in quanto donna non ero adeguata a fare il neurochirurgo e in particolare il chirurgo spinale |
| molestie sessuali |
| Ne ho a mazzi: dal "sei bravina, non fare questo lavoro, che i pazienti non si fideranno mai di te" alle attenzioni moleste da parte di un dirigente, al "Sei brava, ma i tempi non sono ancora maturi per affidare una posizione apicale ad una donna" al commento di un collega: "vorrai mica un posto apicale: hai già avuto la famiglia e fai il tuo lavoro, perchè non ti basta?" Già, però ai maschi non viene mai detto: hai famiglia, non puoi diventare un capo! |
| Negato un incarico in seno a società scientifica b |
| Nessuno |
| Partecipato ad un progetto poi finito sui giornali , i nomi di tutti i miei colleghi compresi gli specializzandi tranne il mio che ero uno dei principali autori ed unica donna . |
| Per i pazienti, purtroppo ancora oggi, le donne sono considerate solo “signore” e “signorine”. |
| Pesanti allusioni sessuali offensive e poco opportune davanti ad altri operatori sanitari ed anche davanti ai pazienti. Altro episodio: quando ero studentessa ed avevo deciso di intraprendere la carriera neurochirurgica, mi è stato detto dal mio Prof universitario che questa "non è una carriera per donna" |
| principalmente non vengo riconosciuta come medico, nè tantomeno come neurochirurgo, dalla popolazione generale in ospedale. pur indossando divisa verde e camice |
| Sono dovuta finire in tribunale contro l’azienda ospedaliera per vedere riconosciuti i diritti di una madre nella cura dei figli sotto i tre anni! Indescrivibile lo sconcerto della relazione di difesa redatta dagli “avvocati” di una pubblica istituzione: denigratoria nei confronti delle donne, del loro lavoro, dei figli e della famiglia per altra avvallata da dei rinomati direttori di Neurochirurgia! |
| Sono ora incinta: ho insistito per lavorare fino al termine della gravidanza perché non credo sia possibile stare lontana dal lavoro per troppi mesi. Ogni giorno tutti mi fanno sentire fuori luogo: le donne nella mia condizione non lavorano fino alla fine dell’allattamento. Avete mai visto un uomo con un lavoro di responsabilità stare fuori dal lavoro per 2 anni? Cosa troverebbe al suo rientro? Inoltre non ho avuto Nessuna agevolazione: ho fatto guardie notturne fino al 5 mese: quando ho chiesto di far rispettare la normativa mi hanno riempito di turni festivi (ovviamente più pesanti rispetto ai feriali). Nessun parcheggio agevolato (come nei supermercati si per esempio). E per il dopo so già che non potrò accedere al nido aziendale: la graduatoria si basa esclusivamente su isee (e non sul ruolo per esempio) pur non avendo tariffe agevolate |
| Sono tanti, dall'epoca della specializzazione. Discriminazione è anche ricevere avances e proposte dai vari strutturati, dai primari, dai Direttori di Scuola di Specializzazione, visto che ai colleghi non succede. Discriminazione è dover mollare la presa, tirare i remi in barca, perché qualunque cosa si faccia in più o meglio di un collega, l'opinione comune è quella di aver ceduto "qualcosa". Discriminazione è essere stata assunta per anni a tempo determinato per aver voluto rinunciare a partecipare un concorso, il cui prezzo della vittoria sarebbe stato troppo alto. Discriminazione è il modo in cui mi trattano i familiari dei pazienti, per i quali una donna al massimo può essere un'infermiera per cui si rivolgono con il tu per chiedere alla "bella signorina" di parlare con il Dottore... Potrei andare avanti all'infinito. E' un mondo terrificante quello della Neurochirurgia. |
| Soprattutto dal punto di vista della carriera. L’avanzamento è riservato solo agli uomini. Quindi ad esempio un giovane con 7 anni meno viene messo in sala operatoria tutti i giorni per un anno perché sia almeno in grado di fare ciò che io già so fare in tutta autonomia così da potergli far fare il salto e sorpassarmi in poco tempo ancora. Tutto sotto la luce del sole senza che io possa far nulla per invertire questo processo. |
| Tanti. Il più recente è stato il posticipare la mia promozione che già tardava a realizzarsi, ma alla data concordata ero incinta perciò l’ho ottenuta solo una volta rientrata dalla maternità nonostante fosse stato concordata tutt’altra tempistica prima ancora di rimanere incinta. |
| un collega più giovane neoassunto mi è stato fatto passare davanti dopo il rientro dalla maternità. Esperienza sicuramente ne ha però io lavoravo li da molto prima e ho pari competenze |

**Domanda 49**

**Hai proposte per migliorare il percorso futuro?**

# 25 risposte

| 1. che la SINCH si faccia promotrice di proposte legislative che favoriscano la condivisione dei carichi familiari, come i congedi di partenità (in Svezia è obbligatoria per 6 mesi, così come per le mamme), la detassazione delle baby sitter etc etc.. 2. promozione di nidi ed asili all'interno delle aziende senitarie (giusto cielo, si tratta di iniziative inventate dagli industriali illuminati dell'Ottocento, possibile che non si riescano ad organizzare negli ospedali degli anni 2000, magari con infermieria annessa, così che papà e mamme non siano costretti ad assentarsi quando i piccoli sono malati). Succede già nella vicina Svizzera, non è fantascienza! che so, magari fornendo un riconoscimento "rosa" alle Aziende Ospedaliere che percorrono queste vie 3. L'istituzione di un Comitato Pari Opportunità (CUG) nella SINCH, che comprenda anche un consulente Legale, che si faccia carico delle segnalazioni di situazioni di discriminazione e supporti le colleghe in difficoltà  4. Niente quote rosa, ma un attento controllo mediante attività di rendicontazione periodica (da parte del CUG) della inclusività con alcuni parametri tipo: la proporzionalità nei ruoli dirigenziali, sia nelle società scientifiche che nei vari dipertimenti NCH, i report di segnalazione e la quota di abbandono da parte delle colleghe |
| --- |
| Aiuti nel caso di maternità e non emarginazione dall’attività operatoria in gravidanza |
| Cambiare la testa alle persone che oggi dirigono la Neurochirurgia Italiana |
| completo ricambio generazionale |
| Difendere la nostra professionalità, difendere la maternità e supportare la famiglia |
| Dovrebbe esserci un cambio di mentalità che solo un cambio generazionale può attuare |
| È necessaria maggiore organizzazione sul posto di lavoro e minore gerarchia professionale |
| Eliminare direttori con mentalità discriminatoria appena si verifica un episodio del genere |
| Eliminare i raccomandati di qualunque sesso. Quella e’ la vera. Piaga italiana |
| Favorire la gestione familiare. Avere un comitato di ascolto |
| Incrementare l’educazione scolastica inserendo dibattiti sulla parità di genere ed il rispetto verso la persona a prescindere dal genere. |
| Inserimento nei concorsi di un punteggio supplettivo per ogni figlio (ogni figlio genera un allontanamento dalla sala operatoria di almeno 12 mesi. Questo va a discapito del curriculum chirurgico) |
| Maggior affiatamento e collaborazione tra donne neurochirurgo |
| Monitoraggio carriera Prima ai giovani nulla era dovuto Ora dopo anni di fatiche contano solo i giovani e solo su di loro si investe e chi non è più giovane viene sacrificato per portare avanti i giovani… Valutare pari opportunità anche in termini di coinvolgimento chirurgico e scientifico ( spesso si viene esclusi dalla sala operatoria o in una carriera chirurgica o da gruppi di ricerca in quanto non destinati da primario alla carriera). Demansionamento ( es RtdA anziché associato quando già in possesso di abilitazione per essere ricattabili, mentre a colleghi maschi viene regalato l’avanzamento di carriera) Mobbing ( saperlo riconoscerlo e poterlo combattere prima di venirne feriti e annientati come professionisti e persone) |
| Nessuna proposta ma l'augurio che possa implodere questo sistema: mi provoca la nausea e mi ha tolto ogni motivazione. L'unica cosa che tiene viva la fiammella è il rapporto con il paziente, è provare ad aiutare chi ha bisogno (e nemmeno più per situazioni mediche). |
| non penso sia migliorabile |
| Obbligare tutte le scuole di specializzazione e tutte le strutture ospedaliere a creare un logbook (che venga confermato sia dal discente che dal docente o dallo strutturato che dal direttore) dove esattamente lo stesso numero di procedure chirurgiche da primo siano esattamente le stesse per uomini e donne, in tutti i loro passaggi. Esempio. Clipping aneurisma: craniotomia eseguita, apertura scissura eseguita, clipping aneurisma eseguito, chiusura eseguita. tutti i passaggi devono essere soddisfatti ed identici per il discente uomo o donna. Altrimenti un discente donna che si è fermato a 2 o 3 dei 4 passaggi e figura come primo op viene sovrapposta al discente uomo che ha fatto tutti e 4 i passaggi. |
| Più tutela per le donne, certificazione della opportunità paritarie ( numero guardie, numero di sale da primo operatore) e tutela nei concorsi/assunzioni per donne in stato di gravidanza o con figli a carico |
| Puntare sull’ educazione |
| Purtroppo no |
| Sensibilizzare le nuove generazioni alla cultura della parità di genere, migliorare le politiche di conciliazione lavoro-famiglia, ridistribuire i carichi di lavoro assistenziale, scientifico in modo equo (ciascuno neurochirurgo dovrebbe avere il diritto e dovere di eseguire un minimo di attività anno (interventi chirurgici/anno, pubblicazioni scientifiche/anni, congressi etc…) |
| Statisticamente più donne fanno medicina, più donne specialità chirurgiche per cui ci saranno più donne in posizioni apicali. Tuteliamo le differenze, lavoriamo bene e speriamo che il welfare nazionale migliori. |
| Tutelare la maternità, rendere fattiva la legge già presente che prevede la sostituzione di chi è in maternità. Questo, per non avere il peso di dover far sobbarcare ai colleghi la propria parte di lavoro. |
| Valorizzare il merito, non il genere. |
| valutazione dei primari totalmente autonoma che preveda anche voci sulla discriminazione. Se valutati male non hanno “bonus”. Con l’incentivo del denaro dovrebbero stare più attenti |
