# Supplementary material for: Women in neurosurgery aim for recognition of merit, not tokenism: insights from an Italian survey
Source: Front Surg. 2025 Jun 2;12:1594731. doi: 10.3389/fsurg.2025.1594731 (PMC12171119; doi:10.3389/fsurg.2025.1594731)
Supplement: Supplementary file 4 [file Table4.docx]

**Question 22**

**How much time have you been absent from work for maternity leave (in total)?**

# 45 responses are reported below

| 1 year |
| --- |
| 10 months |
| 10 months |
| 10 months, for both children, including holidays |
| 11 months |
| 12 months |
| 12 months |
| 12 months |
| 12 months |
| 14 months |
| 14 months |
| 14 months |
| 16 months |
| 18 months |
| 18 months |
| 2 years |
| 2 years for the 2 pregnancies |
| 22 months |
| 24 months for 2 maternity leaves (one of which is still ongoing; I have included the months I will still be absent) |
| 3 years |
| 3 years |
| 3 months |
| 4 months |
| 5 months |
| 5 months |
| 5 months |
| 5 months |
| 5 months for the first child 5 months for twins |
| 6 months |
| 6 months |
| 6 months |
| 6 months for every child |
| 6 months first child 5 months second child |
| 7 months |
| 8 months |
| 9 months |
| Less than a year during the specialization |
| Few months |
| 6 months |
| 6 months |
| 3 months |
| 1 year |
| 1 year |
| 1 month |
| Ongoig |

**Question 40**

**If you have had work experience abroad, where?**

57 had experience abroad at the locations listed below

UK

Cambridge, UK

Leeds, UK

London

Study UK and California

Oxford, UK

UK

Germany

Hannover- Germany

Hannover, Germany

USA

USA

USA

USA

USA

San Francisco

USA

USA

USA

USA, California

Fellowship New York

Los Angeles

Mayo Clinic Rochester, Istanbul, Mexico, Germany

New York

NYC

Strasbourg

France

France

France

France

France

France

France

France, Switzerland, Germany

France

France, Lyon

Lyon

Lyon, London, San Francisco, but for short periods

Montpellier

Paris (1 residency year) and Cincinnati (7 months, research on cadaver)

Paris, Orlando, Alicante

Switzerland

Switzerland

Switzerland

Switzerland

Switzerland and France

Geneva

Geneva, Switzerland

Zurich

East Europa

Helsinki

Malaga

North Europe

Sweden

Toulouse

Mexico

Various places

**Question 48**

**If it has happened to you, briefly describe an episode of discrimination you have experienced:**

37 responses

| Statement from a chief during the hiring process: 'You are 38 years old, you're old, and be grateful for the position (after 24 competitions, I came second or first in 8 years). But you could still get pregnant, so I won't invest in you…' Many others:   - After a competition: 'You’re good, but your colleague has three children, and I’m sorry, I have to hire him, poor guy (me... I wish I could have had children too)...' - 'You’re too young.' - 'You’re too old.' - 'You talk too much' (when, in reality, men always talk). Etc., etc. |
| --- |
| "If you don't have children for at least the next 10 years, you could become a good surgeon." |
| During the specialty exam, the professor asked me why, as a woman, I chose neurosurgery since it is incompatible with having a family.  Even today, PATIENTS ask me to introduce them to a good surgeon. |
| During my second pregnancy, I did not receive a contract renewal and I filed a lawsuit with the Equal Opportunities Committee |
| Removal from the operating room |
| After 5 years as a specialist, I cannot perform a herniated disc surgery on my own, while a newly qualified specialist can do a simple spinal procedure independently. |
| During the first years of specialization, always being disadvantaged compared to male colleagues because I am a woman. |
| During an interview, I was asked if I had plans for a pregnancy. |
| During one of the training periods in the hospital within the training network, a male colleague was given the opportunity to participate in multiple scientific projects. As for me, despite expressing a desire to participate in research activities, I was only given one project to do by the end of the period, and it was even nursing-oriented. |
| It is quite common for patients to be skeptical about my diagnostic and therapeutic conclusions, especially during outpatient visits. They often compare my recommendations with those of a male colleague, trusting his suggestions more than mine. |
| Being called a nurse, being kept out of the operating room because I’m not considered capable enough or too weak for the type of surgery. |
| Being constantly called 'little doctor' by a senior staff member. Always with the same senior staff member in a patient's room, I wearing scrubs and a gown, him in civilian clothes, the patient identified him as the doctor, while I was called 'miss.' The senior staff member's reaction was a loud laugh. These are the episodes that come to mind immediately, but there have certainly been many others. However, I have never faced limitations in the operating room, nor in terms of training activities in general. I commend my chief, who has four women on the team and makes no discrimination! |
| There are too many episodes. |
| I have male colleagues who can take time off for their children, while if a woman does the same, she is criticized and excluded. In my professional life, I have always had to give more than my male colleagues to achieve less. |
| I had to give up maternity leave for breastfeeding in advance because otherwise, access to the operating room would have been difficult for me (possible during service hours after the 7th month postpartum). |
| I have faced more obstacles from an older female neurosurgeon colleague than from male colleagues. Now, the relationship among colleagues is absolutely equal. Moral: sometimes, we can even be our own enemies. |
| The father of a patient, at the end of the surgery, instead of speaking with me, the surgeon, only spoke with the anesthetist because he was a man. |
| The patient asked to speak with 'the doctor,' referring to my male colleague. |
| The chief (Prof. S.) stopped me during the rounds and had them continue with a first-year resident, saying that I lacked the knowledge because I had been absent for a long period (maternity leave, convalescence due to a tumor-related illness). |
| The majority of patients do not perceive me as a neurosurgeon capable of performing surgery. I am often mistaken for a nurse or a healthcare assistant. |
| Missed opportunity to postpone European training course due to pregnancy and breastfeeding. |
| I have definitely been discouraged from starting this career, but once I began, I found people who were supportive and encouraged me a lot. |
| I was advised to terminate my pregnancy to prioritize work. I have been subjected to repeated unsolicited sexual advances. I was told that, as a woman, I was not suited to be a neurosurgeon, especially a spinal surgeon. |
| Sexual harassment |
| I have plenty: from 'You're good, but don't do this job, the patients will never trust you,' to unwanted attention from a manager, to 'You're good, but the time isn't right yet to give a leadership position to a woman,' to a colleague's comment: 'You wouldn't want a leadership position, you've already had a family and you do your job, why isn't that enough for you?' Yeah, but men are never told: 'You have a family, you can't become a leader! |
| Denial of a position within a scientific society |
| None |
| I participated in a project that later made it to the newspapers, with the names of all my colleagues, including the residents, except mine, even though I was one of the main authors and the only woman. |
| For patients, unfortunately, even today, women are still considered only 'ladies' and 'misses.' |
| Offensive and inappropriate sexual innuendos in front of other healthcare workers and even in front of patients. Another incident: when I was a student and had decided to pursue a career in neurosurgery, my university professor told me that this 'is not a career for a woman. |
| Mainly, I am not recognized as a doctor, let alone as a neurosurgeon, by the general population in the hospital, even while wearing green scrubs and a white coat. |
| I had to end up in court against the hospital management to have a mother's rights in caring for children under three years old recognized! The shock of the defense report written by the 'lawyers' of a public institution is indescribable: it was denigratory towards women, their work, their children, and their families, and further supported by renowned directors of Neurosurgery! |
| I am now pregnant: I insisted on working until the end of my pregnancy because I don't believe it's possible to be away from work for too many months. Every day, everyone makes me feel out of place: women in my condition don't work until the end of breastfeeding. Have you ever seen a man in a responsible job stay away from work for 2 years? What would he find when he returns? Also, I haven’t had any special accommodations: I did night shifts until the 5th month. When I asked for the regulations to be respected, they filled me with holiday shifts (which, of course, are heavier than regular weekday shifts). No special parking (like in supermarkets, for example). And for the future, I already know I won’t be able to access the company daycare: the ranking is based solely on the ISEE (income index), not on the role, for instance, even though there are no discounted rates. |
| There are many, dating back to the time of my residency. Discrimination is also receiving advances and proposals from various senior staff, chiefs, and Directors of the Residency School, since this doesn't happen to my male colleagues. Discrimination is having to let go, pull back, because whatever you do that's more or better than a colleague, the common opinion is that you've 'given up something.' Discrimination is having been employed for years on a fixed-term contract because I wanted to refuse to participate in a competition, the price of which for victory would have been too high. Discrimination is the way I am treated by the patients' families, for whom a woman can at most be a nurse, addressing me informally to ask the 'pretty young lady' to speak with the Doctor... I could go on forever. The world of Neurosurgery is a terrifying one. |
| Especially from a career perspective. Advancement is reserved only for men. For example, a young man who is 7 years younger is put in the operating room every day for a year to be at least able to do what I already know how to do completely independently, so that he can make the leap and surpass me in a short time. All of this happens openly, with no way for me to reverse this process. |
| Many. The most recent one was the postponement of my promotion, which was already delayed, but by the agreed-upon date, I was pregnant, so I only received it once I returned from maternity leave, even though a completely different timeline had been agreed upon before I became pregnant. |
| A younger, newly hired colleague was put ahead of me after I returned from maternity leave. He certainly has experience, but I had been working there much earlier and have equal competencies. |

**Question 49**

**Do you have any suggestions for improving the future path?**

# 25 responses

| 1. That the SINCH promote legislative proposals that encourage the sharing of family responsibilities, such as paternity leave (in Sweden, it is mandatory for 6 months, as it is for mothers), tax exemptions for babysitters, etc. 2. Promotion of nurseries and daycares within healthcare organizations (indeed, these initiatives were invented by enlightened industrialists in the 19th century, why can’t they be organized in 2000s hospitals, possibly with attached nursing services, so that fathers and mothers don’t have to take time off when their children are sick). This already happens in neighboring Switzerland, it’s not science fiction! For example, providing a “pink” recognition for hospital organizations that pursue these initiatives. 3. The establishment of an Equal Opportunities Committee (CUG) within SINCH, which includes a Legal Advisor to handle reports of discrimination and support colleagues in difficulty. 4. No gender quotas, but careful monitoring through periodic reporting (by the CUG) on inclusivity with parameters such as: proportionality in leadership roles, both in scientific societies and in various NCH departments, reporting data, and the rate of female attrition. |
| --- |
| Assistance in the case of maternity and not being marginalized from surgical activities during pregnancy. |
| Change the mindset of the people who currently lead Italian Neurosurgery. |
| Complete generational turnover. |
| Defend our professionalism, defend maternity, and support the family. |
| There should be a change in mindset that only a generational shift can bring about. |
| Greater organization in the workplace and less professional hierarchy are needed. |
| Eliminate directors with discriminatory mindsets as soon as such an incident occurs. |
| Eliminate the favored individuals of any gender. That is the true Italian plague. |
| Promote family management. Have a listening committee. |
| Increase school education by including debates on gender equality and respect for individuals regardless of gender. |
| Inclusion in competitions of an additional score for each child (each child results in at least 12 months away from the operating room, which negatively impacts the surgical curriculum). |
| Greater camaraderie and collaboration among female neurosurgeons. |
| Career monitoring. Previously, nothing was expected of the young. Now, after years of hard work, only the young matter, and all investment is directed at them, while those who are no longer young are sacrificed to make way for the younger generation… Evaluate equal opportunities also in terms of surgical and scientific involvement (often, individuals are excluded from the operating room or from a surgical career or research groups because they are not designated by the senior surgeon for such a career). Demotion (e.g., RtdA instead of associate, even when already qualified, to make them vulnerable to blackmail, while male colleagues are given career advancement). Mobbing (knowing how to recognize it and being able to fight it before being harmed and destroyed as professionals and individuals). |
| No proposals, but the wish that this system might implode: it makes me sick and has taken away all my motivation. The only thing that keeps the flame alive is the relationship with the patient, trying to help those in need (and not even for medical situations anymore). |
| I don't think it can be improved. |
| Require all specialty schools and hospital facilities to create a logbook (confirmed by both the trainee and the instructor or senior staff member as well as the director) where the exact same number of surgical procedures performed as the primary surgeon are identical for both men and women, in all their steps. Example: Aneurysm clipping: craniotomy performed, fissure opened, aneurysm clipped, closure performed. All steps must be completed and identical for male or female trainees. Otherwise, a female trainee who has completed 2 or 3 of the 4 steps and is listed as the primary surgeon would be compared to a male trainee who has completed all 4 steps. |
| More protection for women, certification of equal opportunities (number of on-call shifts, number of first-surgeon operating rooms) and protection in job competitions/appointments for women who are pregnant or have children to care for. |
| Emphasize education. |
| Unfortunately, no. |
| Raise awareness among new generations about the culture of gender equality, improve work-life balance policies, and redistribute caregiving and scientific workloads fairly (every neurosurgeon should have the right and duty to perform a minimum amount of activities per year, such as surgical procedures per year, scientific publications per year, conferences, etc.). |
| Statistically, more women are entering medicine, and more women are choosing surgical specialties, so there will be more women in leadership positions. Let’s protect the differences, work well, and hope that national welfare improves. |
| Protect maternity leave by making the existing law effective, which provides for the replacement of those on maternity leave. This is to avoid the burden of having colleagues take on your share of the work. |
| Value merit, not gender. |
| Evaluation of department heads should be completely independent and include aspects related to discrimination. If they are evaluated poorly, they should not receive any "bonuses." With the financial incentive, they should be more attentive. |
